# Comparative transcriptomic analysis of the evolution and development of flower size in *Saltugilia* (Polemoniaceae)

**DOI:** 10.1186/s12864-017-3868-2

**Published:** 2017-06-23

**Authors:** Jacob B. Landis, Douglas E. Soltis, Pamela S. Soltis

**Affiliations:** 1 Department of Biology, University of Florida, Gainesville, FL 32611 USA; 2 Florida Museum of Natural History, University of Florida, Gainesville, FL 32611 USA; 3 Department of Botany and Plant Sciences, University of California Riverside, 4412 Boyce Hall, 3401 Watkins Drive, Riverside, CA 92521 USA; 4 Genetics Institute, University of Florida, Gainesville, FL 32610 USA

**Keywords:** RNA-Seq, Exon capture, Flower Evo-Devo, Corolla length, Differential expression

## Abstract

**Background:**

Flower size varies dramatically across angiosperms, representing innovations over the course of >130 million years of evolution and contributing substantially to relationships with pollinators. However, the genetic underpinning of flower size is not well understood. *Saltugilia* (Polemoniaceae) provides an excellent non-model system for extending the genetic study of flower size to interspecific differences that coincide with variation in pollinators.

**Results:**

Using targeted gene capture methods, we infer phylogenetic relationships among all members of *Saltugilia* to provide a framework for investigating the genetic control of flower size differences via RNA-Seq de novo assembly. Nuclear concatenation and species tree inference methods provide congruent topologies. The inferred evolutionary trajectory of flower size is from small flowers to larger flowers. We identified 4 to 10,368 transcripts that are differentially expressed during flower development, with many unigenes associated with cell wall modification and components of the auxin and gibberellin pathways.

**Conclusions:**

*Saltugilia* is an excellent model for investigating covarying floral and pollinator evolution. Four candidate genes from model systems (*BIG BROTHER, BIG PETAL, GASA*, and *LONGIFOLIA*) show differential expression during development of flowers in *Saltugilia*, and four other genes (*FLOWERING-PROMOTING FACTOR 1*, *PECTINESTERASE, POLYGALACTURONASE,* and *SUCROSE SYNTHASE*) fit into hypothesized organ size pathways. Together, these gene sets provide a strong foundation for future functional studies to determine their roles in specifying interspecific differences in flower size.

**Electronic supplementary material:**

The online version of this article (doi:10.1186/s12864-017-3868-2) contains supplementary material, which is available to authorized users.

## Background

Evolutionary developmental biology, Evo-Devo, aims to understand the origins of morphological variation, which may be caused by variation in genomes or environmental interactions during development [1, 2]. The evolution of similar traits in distinct lineages is often associated with allelic changes in a particular gene [3], with differential regulation of expression of these genes frequently associated with speciation events or in response to environmental and ecological pressures [4, 5]. Early studies in Evo-Devo relied on comparisons of single candidate genes or a single gene network resulting from a priori knowledge from model systems, which may be distantly related to the species of interest [6, 7]. The advent of transcriptome sequencing has led to an increased push in analysis of non-model species, including fish [8], invertebrates [9, 10], mammals [11], and land plants [12]. The number of studies using gene expression to investigate natural variation, responses to stimuli, and the roles that differential gene expression plays in phenotypes is on the rise [13]. Transcriptome-based investigations into aspects of floral evolution in groups without a sequenced genome have addressed the genetics of sex-specific flowers [14], profiling of outcrossing vs. selfing flowers [15, 16], flower scent [17], pistillate flowering [18], and gene regulation in floral pathways [19–21].

These de novo approaches are ideal for studies involving the phlox family (Polemoniaceae). Currently there are no genome-level resources available for any members of Polemoniaceae, and only two studies have been published using transcriptomic data: one on a single species of *Phlox* [22] and the other on two species of *Saltugilia* [23]. Comparative studies focused on closely related species in Polemoniaceae have shown drastic differences in flower size in species of *Ipomopsis* [24], *Leptosiphon* [25], and *Saltugilia* [23]. One to six QTL were identified in *Ipomopsis* for traits such as stamen length, pistil length, corolla tube length, corolla tube width, and anthocyanin abundance [24]. The genetics of organ size in plants, especially flower size, are largely unknown. However, candidate genes associated with flower size differences have been identified in model systems, predominantly *Arabidopsis* [26–35]. The hypothesized gene networks of many, but not all, of these genes have been reviewed and outlined [35, 36], but work remains, particularly in non-model species, to clarify if – and how – these genes determine flower size.

**Fig. 1:**
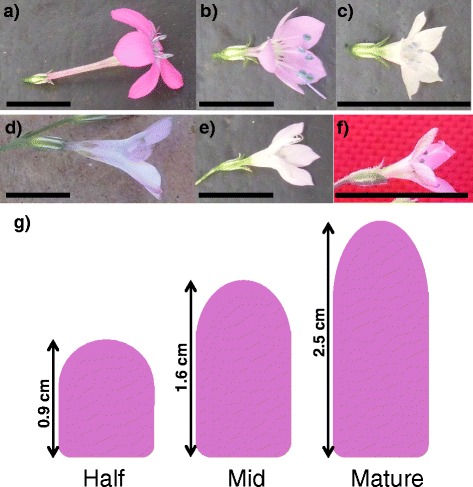
Representatives of flowers of *Saltugilia*. **a** *S. splendens* subsp. *grantti,* **b** *S. caruifolia,* **c** *S. australis,* **d** *S. splendens* subsp. *splendens* (FS), **e** *S. splendens* subsp. *splendens* (GH), **f** *S. latimeri*, **g** schematic of the different stages of development sampled in *S. splendens* subsp. *grantii*. All scale bars equal 1 cm

Based on previous analyses of *Saltugilia* [23], the roles of cell size and cell number in generating differences in flower size are well established, with differences in cell size playing a greater role than changes in cell number. The observed differences in flower length are also associated with changes in reproductive behavior, with the small-flowered species being autogamous and the large-flowered species pollinated by hummingbirds and bee flies [37, 38]. Despite the potential of *Saltugilia* for addressing questions of floral Evo-Devo and associated variation due to pollinators, the phylogenetic relationships among species have been difficult to resolve, with multiple studies having inferred different relationships among members [23, 39]. These difficulties likely stem from hybridization events between taxa, with both Grant and Grant [40] and Weese and Johnson [38] showing taxa to be interfertile and capable of producing vigorous hybrids. Members of *Saltugilia* exhibit a 2.5-fold change in flower size (Fig. 1a-f), as well as differences in observed pollinators. The small-flowered taxa, *S. australis* and *S. latimeri*, possess corollas 0.8 cm long, while the large-flowered taxa, *S. splendens* subsp. *grantii* and *S. splendens* subsp*. splendens*, have flowers 2.5 cm and 2.3 cm long, respectively. Thus, further analysis is required to provide the necessary context for interpreting changes in flower size and associated shifts in gene expression and pollinators.

The goals of this study are two-fold: first, we investigate phylogenetic relationships within *Saltugilia* using current analytical methods for next-generation sequencing data, as well as a comparison between concatenation and species tree inference methods; second, we investigate patterns of differential gene expression associated with differences in flower size in representatives of the six taxa of *Saltugilia* (*S. australis, S. caruifolia, S. latimeri, S. splendens* subsp. *grantii*, *S. splendens* subsp. *splendens* (GH; denoting that this sample was grown in the greenhouse common garden), and *S. splendens* subsp. *splendens* (FS; denoting that this is plant material collected in the field)) using RNA-Seq methods. Specifically, we identify differentially expressed genes (1) throughout floral development in each taxon, (2) throughout floral development across all taxa, and (3) between small- and large-flowered species.

## Methods

### Plant material

Four taxa of *Saltugilia* and one taxon of *Gilia* were grown in the greenhouses at the University of Florida from seeds: *G. brecciarum* subsp. *brecciarum*, *S. australis, S. caruifolia*, *S. splendens* subsp. *grantii*, and *S. splendens* subsp. *splendens* (GH). Additionally *G. stellata*, *G. brecciarum*, *S. latimeri*, and *S. splendens* subsp. *splendens* (FS) were collected in California in the spring of 2015 with the help of Tasha LaDoux (University of California Riverside, USA). Finally, three accessions of *Gymnosteris, G. nudicaulis* and *G. parvula* (two accessions)*,* were sampled from herbarium material (Additional file 1: Table S1).

### Phylogenetic analysis

A detailed description of the targeted gene capture method for phylogenetic analysis has previously been described [23]. Cleaned sequencing reads were used for 10 accessions spanning *Gilia* and *Saltugilia* to infer phylogenetic relationships, with three accessions of *Gymnosteris* used as outgroups (Additional file 1: Table S1). Briefly, MYbaits (MYcroarray; Ann Arbor, MI, USA) probes for 100 putatively single-copy nuclear genes were designed from reciprocal BLAST of four transcriptomes of closely related taxa (*Fouqueria macdouglaii, Phlox drummondii, Phlox* sp.*,* and *Ternstroemia gymnanthera*) available in the 1KP data set (www.onekp.com) using MarkerMiner [41] to identify single-copy genes. Selected genes were chosen to represent all of the chromosomes in *Arabidopsis* and to contain large exon regions with few, small intronic regions. Capture products were sequenced on three independent Illumina runs and analyzed using HybPiper [42]. On-target reads of nuclear genes and by-product plastid reads were assembled to individual gene references using default parameters following Burrows-Wheeler Aligner analyses [43]. Contigs for each gene from all taxa were aligned using MAFFT (version 7.245; [44]) installed on the University of Florida Research Computing cluster using pairwise comparisons with 1000 iterations.

Phylogenetic analysis of assembled loci followed two alternative approaches: concatenation of genes (nuclear and plastid genes separately) and species tree inference using nuclear genes. For the concatenation analysis, aligned gene sequences with at least 450 bp of overlapping sequence were concatenated into a single file using SequenceMatrix [45]. Separate concatenated matrices of 54 nuclear genes and 80 plastid protein-coding genes were then analyzed using PartitionFinder (version 2.0; [46]) using a greedy algorithm and RAxML (version 8; [47]) to find the best partitioning scheme for each matrix. The maximum-likelihood tree for each matrix was inferred using RAxML and the previously identified partitions, with an initial 1000 bootstrap replicates before beginning the search for the best tree for each matrix. Because the nuclear and plastid trees differed in topologies (see below), these data were not combined into a single analysis.

Species tree inference was performed in Astral [48] using the same 54 nuclear genes used for the concatenation analysis. Topologies for individual gene trees were inferred using RAxML with a GRT + G model of evolution. One thousand bootstrap replicates were conducted for each locus, and the inferred maximum-likelihood tree was used for species tree inference. Support for the inferred species tree topology was obtained by running 500 multi-locus bootstrap replicates.

### RNA-Seq library preparation and sequencing

Total RNA was extracted from whole flowers using Tri-Reagent following the manufacturer’s directions (Ambion, Austin, TX, USA) from the three developmental stages: half stage (flowers 50% the length of flowers at anthesis), mid stage (flowers 75% the length of flowers at anthesis), and mature stage (directly after anthesis) (Fig. 1g). These developmental stages were the same stages previously used for characterizing cell number and cell size through floral development in *Saltugilia* [23]. Flowers were collected from two individuals from the same population except for *S. latimeri*, in which only one flower of the half stage was collected because a second was not available at the time of collection.

Quality of total RNA for each sample was determined using an Agilent 2100 Bioanalyzer (Agilent Technologies, Santa Clara, CA, USA) at the University of Florida Interdisciplinary Center for Biotechnology Research (ICBR). Library preparation was performed using the NEBNext Ultra RNA Library Prep Kit for Illumina (NEB, Ipswich, MA, USA) following the manufacturer’s directions and NEBNext Multiplex Oligos for Illumina Index Primers Set 1 and 2 barcodes (NEB, Ipswich, MA, USA). Transcriptome sequencing was performed at the University of Florida ICBR using a single run on the Illumina NextSeq 500 (2 × 75 bp). The desired sequencing coverage was approximately 30 million reads per sample, but fewer reads were acceptable, given that greater replication has been found to be more beneficial for investigations of differential gene expression than sequencing depth [49]. Raw reads were trimmed and filtered using the pipeline described previously [23] using Cutadapt [50] and Sickle [51], with modifications to the quality parameter (minimum quality score of 26) and length parameter (minimum length of 35 bp) for each read that passed the filtering criteria.

### Transcriptome analysis

A de novo approach using Trinity [52, 53] was followed, given that no published genome is available in Polemoniaceae. All reads for each taxon were consolidated into a single file prior to in silico normalization to reduce complexity of reads by reducing the most abundant reads to 50× before concatenation with other taxa to generate a master reference assembly. This method reduces the total number of transcripts formed during assembly, thus likely making comparisons of differential expression more robust. The non-normalized read count for each taxon ranged from 43,474,704-139,864,452 paired-end reads (Table 1). Concatenation of the normalized reads resulted in 103,525,951 paired-end reads used for de novo assembly of a reference assembly. Non-normalized sequencing reads were then mapped back to the assembled reference transcriptome using RNA-Seq by Expectation-Maximization as implemented with bowtie2 [54] and filtered at the 1 TPM (Transcripts Per Kilobase Million) level to reduce false discoveries [55]. These newly created fasta files were translated into open reading frames and translated to protein sequences using the Transdecoder plugin in Trinity for absence/presence comparisons using OrthoVenn [56]. OrthoVenn was also used to assign putative orthology with its implementation of OrthoMCL [57]. Initial analyses included all six taxa, but a more conservative approach was also undertaken in which all samples of *S. caruifolia* and *S. splendens* subsp. *splendens* (GH) were removed from further comparisons due to issues with sequencing coverage and estimated number of protein clusters representing transcriptome size (see below).

**Table 1:** Summary of the quality of the transcriptome for each taxon, including percent of cleaned reads mapped, Trinity genes, total transcripts, percent GC, N50, median contig length, average contig length, and total assembled bases

	*Saltugilia australis*	*Saltugilia caruifolia*	*Saltugilia splendens* subsp. *grantii*	*Saltugilia latimeri*	*Saltugilia splendens* subsp. *splendens* (GH)	*Saltugilia splendens* subsp. *splendens* (FS)
Percent Mapped	74.16	72.18	73.32	80.88	61.05	80.39
Total Trinity genes	92,672	51,020	63,625	63,057	69,371	62,667
Total Trinity transcripts	110,965	71,296	84,178	91,574	92,540	92,544
Percent GC	44.79	45.44	45.64	45.5	44.71	45.5
N50	446	446	547	668	467	661
Median contig length	263	303	320	345	319	341
Average contig length	407.51	424.47	483.93	544.76	432.03	540.55
Total assembled bases	45,219,574	30,263,050	40,736,482	49,885,494	39,980,145	50,024,785

The generated master reference fasta file was annotated using the Trinotate pipeline by performing a BLASTx analysis against the Swiss-Prot database [58] to generate Gene Ontology (GO) terms, as well as KEGG pathways using the EggNOG database [59]. The GO terms generated with the BLASTx analysis were imported into CateGOrizer [60] to categorize and visualize into three broad biological terms using the Plant GO-Slim database: biological processes, molecular functions, and cellular components.

Differential expression analyses were conducted using the Bioconductor DESeq2 package [61] with a *p*-value cutoff of 0.05 and a four-fold difference in expression. Only DESeq2 was used given that Rapaport et al. [62] showed good concordance with this method and other methods such as edgeR [63] and CuffDiff [64]. Differential expression comparisons within each taxon were performed among the development stages with two biological replicates for each stage.

Comparisons of the same developmental stage (e.g. half, mid, mature) among all taxa were conducted by pooling all taxa from a single stage together as biological replicates. However, based on the low sequencing coverage in samples of *S. caruifolia* and *S. splendens* subsp. *splendens* (GH) where many accessions were well below the targeted 30 million reads*,* a more conservative approach was undertaken by removing these taxa completely from further analyses. Removal of *S. caruifolia* and *S. splendens* subsp. *splendens* (GH) left eight biological replicates per sample for the mid and mature stages and seven biological replicates for the half stage. This comparison was deemed to have more power to identify differentially expressed genes due to the increased number of biological replicates [49]. Lastly, comparisons between the mature stages of the small-flowered *S. australis* and *S. latimeri* against the mature stages of the large-flowered *S. splendens* subsp. *grantii* and *S. splendens* subsp. *splendens* (FS) were conducted to identify genes that may be associated with observed differences in flower size. Classification as either small-flowered or large-flowered is solely based on relative corolla length, as can be seen in Fig. 1. The half stages were not included in this analysis due to only having one accession of *S. latimeri*, and the mid stages were not included because this stage did not show many differentially expressed genes in developmental analyses within each taxon.

## Results

### Targeted capture efficiency and phylogenetic analyses

Of the 100 single-copy nuclear genes for which probes were designed, 94 were sequenced in at least one taxon, with 49 genes captured for all taxa. Average contig length for each gene ranged from 29 bp to 2195 bp. Additionally, 80 protein-coding genes of the plastid genome were sequenced as by-product in all 13 samples, with mean contig lengths ranging from 93 bp to 6816 bp (Additional file 2: Table S2). After MAFFT alignments were completed for each gene, 54 nuclear and 80 plastid protein-coding genes exhibited sufficient overlap (i.e. were captured from at least 7 of the 13 samples) for analysis, spanning the six taxa of interest and the associated outgroups. The final concatenated nuclear data set consisted of 58,318 bp of data, with a data matrix exhibiting only 20.67% missing data. The plastid data set consisted of 64,573 bp of data with only 1.53% missing data.

**Fig. 2:**
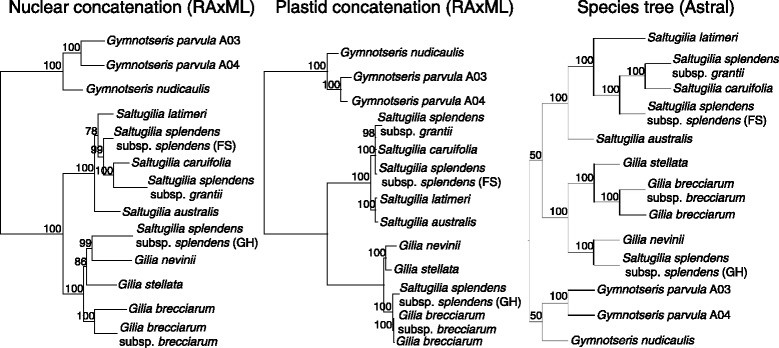
Maximum-likelihood phylogenetic inference of *Saltugilia*, with additional sampling of *Gilia* and *Gymnosteris,* for both nuclear and plastid protein-coding genes. Also included is the species tree inference using nuclear genes. Support values from 1000 bootstrap replicates are shown for concatenated nuclear and plastid genes, and support values of 500 bootstraps for species tree inference are shown

The species tree inference resulted in a topology identical to the concatenated nuclear phylogeny (Fig. 2). The only differences between the two phylogenies are in the support values for the *Gymnosteris* clade and the clade of *Gilia/Saltugilia*. In the concatenated nuclear tree, both clades have bootstrap support values of 100%. In the species tree, these clades have bootstrap support of only 50%. The remaining relationships in the species tree have bootstrap support values of 100%, similar to values in both the concatenated nuclear and plastid phylogenies. The inferred relationships among taxa differ slightly between trees based on the nuclear (both concatenated and species tree) and plastid analyses (Fig. 2). The three accessions of *Gymnosteris* form a well-supported, monophyletic group. The four samples of *Gilia* plus *S. splendens* subsp. *splendens* (GH) form a well-supported clade. However, the closest relative of *S. splendens* subsp. *splendens* (GH) differs between the nuclear and plastid trees, with *G. nevinii* supported as its sister in the nuclear trees and the two accessions of *G. brecciarum* supported as its sister in the plastid tree. All accessions of *Saltugilia* except *S. splendens* subsp. *splendens* (GH) form a well-supported clade, although the relationships among taxa differ. The nuclear trees from concatenation and species tree reconciliation show *S. australis* and *S. latimeri* sister to the rest of *Saltugilia*, while in the plastid tree, these two species form a clade and are sister to the rest of the genus. In both nuclear and plastid trees, *S. splendens* is polyphyletic, with *S. splendens* subsp. *grantii* sister to *S. caruifolia* and *S. splendens* subsp. *splendens* (FS) sister to these two taxa.

### Transcriptome analyses

The unfiltered, assembled master de novo reference compiled from data from all six taxa consisted of 969,222 Trinity genes (or clusters of isoforms that share significant overlap) and 1,222,113 total transcripts (Trinity genes + isoforms), with an N50 of 383 bp and an average contig size of 387 bp. For each taxon, 61.05-80.88% of all reads mapped back to the master reference assembly, resulting in assemblies containing 71,296 to 110,965 transcripts filtered at the 1 TPM level representing the full transcriptome of that taxon (Table 1) and an N50 value ranging between 446 and 668 bp. Individual developmental stages varied in percent of reads mapping to the reference, ranging from 50.19 to 85.74%, with all six samples of *S. splendens* subsp. *splendens* (GH) mapping at 65% or lower (Additional file 3: Table S3), substantially lower than the majority of samples. The lowest percent mapped was the mature stage of *S. caruifolia* plant 4, which also contained the fewest reads that passed the cleaning criteria. The total number of transcripts for each developmental stage also varied, ranging from 41,774 to 136,467 transcripts (Additional file 3: Table S3). The annotation analyses resulted in 23.1% of all transcripts reporting a Swiss-Prot top BLASTx hit, 18.0% having a functional annotation from the EggNOG database, and 23.4% annotated with GO terms of known function (Dryad doi:10.5061/dryad.mc270). Of the annotated GO terms, 16.8% were molecular function genes with 1178 unique terms, 12.30% were cellular component genes with 859 unique terms, and 2.5% were biological process genes with 179 unique terms.

**Fig. 3:**
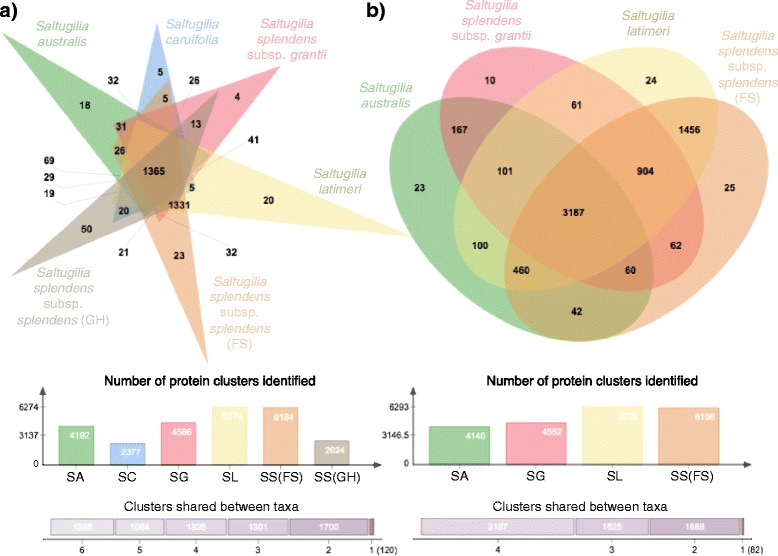
Venn diagrams generated by OrthoVenn [56] showing the number of shared and unique protein clusters for each taxon. **a** Comparison of all six taxa. **b** Comparison following a more conservative approach with removal of *S. caruifolia* and *S. splendens* subsp. *splendens* (GH) due to low read coverage

Comparison of the filtered, translated transcriptomes of the six taxa resulted in a total of 6876 clusters of proteins identified, with 6756 putatively orthologous clusters and 1104 single-copy gene clusters by OrthoVenn’s [56] implementation of OrthoMCL [57]. The number of clusters for each taxon varied: *S. australis* (4192), *S. caruifolia* (2377), *S. latimeri* (6274), *S. splendens* subsp. *grantii* (4586), *S. splendens* subsp. *splendens* (GH) (2624), and *S. splendens* subsp. *splendens* (FS) (6184) (Fig. 3a). In all, 1365 clusters are shared among all six taxa, with three of these shared clusters GO-enriched (based on a hypergeometric test performed with OrthoVenn): unsaturated fatty acid biosynthetic process, alternative mRNA splicing via spliceosome, and mitochondrial respiratory chain complex III. The two taxa collected in the field, *S. latimeri* and *S. splendens* subsp. *splendens* (FS), share 1331 clusters of proteins, more than any other comparisons between taxa. In these samples, five clusters show GO enrichment: regulation of gene expression, lateral root morphogenesis, NAD+ ADP-ribosyltransferase activity, regulation of RNA biosynthetic process, and cellular response to light stimulus.

The more conservative approach involving the removal of both *S. caruifolia* and *S. splendens* subsp. *splendens* (GH) produced a total of 6682 clusters, with 6600 defined as putatively orthologous, and an additional 2712 single-copy gene clusters (Fig. 3b). Individual taxon clusters were similar to the previous analysis, with *S. australis* containing 4140, *S. latimeri* 6293, *S. splendens* subsp. *grantii* 4552, and *S. splendens* subsp. *splendens* (FS) 6196 clusters. Almost half of all clusters were shared among the four taxa (3187), with the two field samples again sharing more clusters than any other pair of taxa (1456). The clusters shared among all four taxa did not show any enriched GO categories, while the shared clusters in the two field samples show four enriched terms: lateral root morphogenesis, NAD+ ADP-ribosyltransferase activity, regulation of gene expression, and response to other organisms.

The large-flowered taxa, *S. splendens* subsp. *grantii* and *S. splendens* subsp. *splendens* (FS), share 62 protein clusters, with 20 showing enriched GO terms involved with activity associated with nutrients or elements (e.g. sulfur dioxygenase activity, nitric oxide biosynthetic process, cellular response to azide, etc.). One enriched GO term, tubulin complex, shows a possible direct link to larger flowers with known functions in microtubule formation [65].

### Differential expression

Each of the six taxa showed transcripts differentially expressed throughout floral development, ranging from four to 10,368 transcripts across the three developmental stages: *S. australis* (652), *S. caruifolia* (181), *S. latimeri* (1032), *S. splendens* subsp. *grantii* (4), *S. splendens* subsp. *splendens* (GH) (973), and *S. splendens* subsp. *splendens* (FS) (10,368) (Additional file 4: Table S4). Of the five taxa that included all three stages, *S. australis* and *S. splendens* subsp. *splendens* (FS) show the mature stage being more similar to the mid stage than either is to the half stage by clustering transcript expression levels (Additional file 5: Fig. S1). In two other taxa, *S. splendens* subsp. *grantii* and *S. splendens* subsp. *splendens* (GH), the mid and half stages are more similar to each other than either is to the mature stages. In *S. caruifolia*, the two samples from the mid and mature stages are not identified as being most similar, likely due to insufficient reads for these accessions.

Very few, if any, of the transcripts that were differentially expressed through development within a species were in the broad category of biological process, with the majority of differentially expressed transcripts consisting of cellular component genes (Additional file 4: Table S4). Based on the cellular profile throughout development of flowers in *Saltugilia* [23], extra attention was focused on genes associated with maintenance and modification of the cell wall, as well as auxin and gibberellin hormone pathways. In *S. australis*, three unigenes associated with the cell wall are upregulated in the mature flowers compared with the half-stage flowers, and three unigenes are upregulated in mature flowers compared with mid-stage flowers, with two of the unigenes always showing upregulation in mature flowers compared to any other stage. Flowers of *S. caruifolia*, *S. splendens* subsp. *grantii,* and *S. splendens* subsp. *splendens* (GH) show one unigene associated with the cell wall upregulated in mature flowers compared with half-stage flowers. In *S. latimeri*, five unigenes associated with the cell wall and one unigene associated with auxins are upregulated in mature flowers compared to half-stage flowers.

The taxon with the most upregulated unigenes, *S. splendens* subsp. *splendens* (FS)*,* shows 21 unigenes associated with the cell wall, four auxin-activated signaling genes, 22 unigenes encoding auxin-induced proteins, and four unigenes associated with gibberellins upregulated in mature flowers compared to half-stage flowers. Comparisons between mature and mid flowers show six unigenes associated with the cell wall and one encoding an auxin-induced protein upregulated in mature flowers. All differentially expressed transcripts, along with the top BLASTx hit, EGGnog annotation, and the top GO category are found in Additional file 6: Table S5.

Of all the differentially expressed unigenes identified through development within taxa, 28 unigenes, including two biological process genes, eight cellular component genes, four molecular function genes, and 14 unannotated transcripts, are differentially expressed in the mature stages compared to the mid stage in at least two taxa. One gene, a cell wall-associated gene with the top BLASTx hit of *POLYGALACTURONASE*, is upregulated in all taxa except *S. splendens* subsp. *splendens* (GH). In comparisons of half-stage and mature flowers in all taxa, 47 unigenes, including 10 cellular component, 11 molecular function, and 26 unannotated unigenes, are upregulated in at least two taxa. One unigene, with the top BLASTx hit of *Tyrosine/DOPA decarboxylase*, is upregulated in all four taxa that show differential expression between the half-stage and mature flowers. Three unigenes, including the cell wall gene *POLYGALACTURONASE*, a molecular function gene with a top BLASTx hit of *1-aminocyclopropane-1-carboxylate oxidase,* and another unannotated gene, are upregulated in three of the four taxa.

**Fig. 4:**
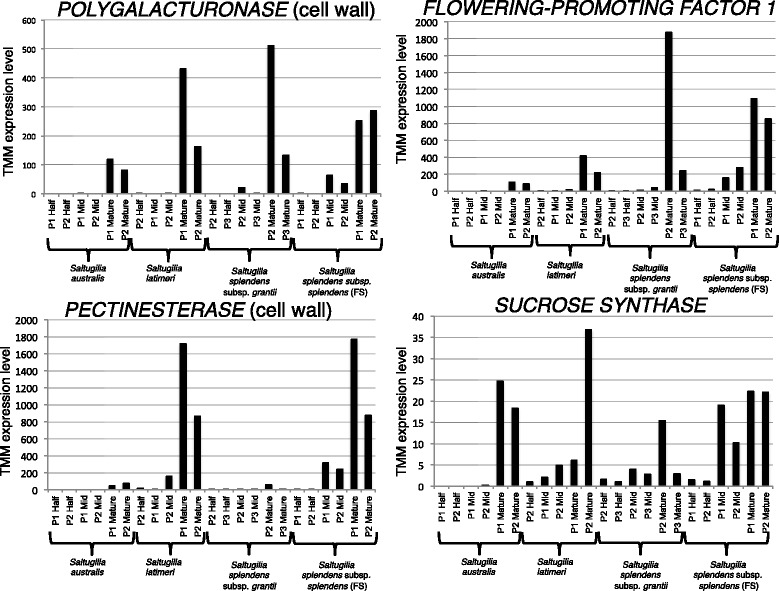
TMM expression levels of four genes, *POLYGALACTURONASE* (cell wall)*, FLOWERING-PROMOTING FACTOR 1, PECTINESTERASE* (cell wall)*,* and *SUCROSE SYNTHASE* showing differential expression among stages of development in *S. australis, S. latimeri, S. splendens* subsp. *grantii*, and *S. splendens* subsp. *splendens* (FS)

Comparisons of the three developmental stages (half, mid, and mature) (Fig. 1g), with each accession representing a biological replicate, resulted in no transcripts differentially expressed between the half and mid stages of development, 45 transcripts differentially expressed between mid and mature stages, and 784 transcripts differentially expressed between half and mature stages. None of the differentially expressed transcripts between the mid and mature stages are associated with cell wall formation or maintenance, or with auxin or gibberellin pathways. However, comparisons between half and mature stages resulted in four unigenes associated with cell wall formation, three with auxin-activated signaling pathways, three with auxin-induced proteins, and two with gibberellin activity. Comparing the differentially expressed unigenes found in mature flowers with those of half-stage flowers, both within and among taxa, resulted in 15 shared unigenes (Table 2). Four of these are likely directly tied to differences in flower size based on their annotations and how they fit into hypothesized organ size pathways [35]: two cell wall unigenes (*POLYGALACTURONASE* and *PECTINESTERASE*), a molecular function transcript (*SUCROSE SYNTHASE*), and a positive regulator of flower development (*FLOWERING-PROMOTING FACTOR 1*) (Fig. 4). *PECTINESTERASE* appears to be upregulated in the two field samples, *S. latimeri* and *S. splendens* subsp. *splendens* (FS), while *FLOWERING-PROMOTING FACTOR 1* appears to be upregulated in the large-flowered species, *S. splendens* subsp. *grantii* and *S. splendens* subsp. *splendens* (FS), than in the small-flowered species, *S. australis* and *S. latimeri*.

**Table 2:** Fifteen unigenes that were shared in comparisons both within and among taxa for different developmental stages, including the Trinity Gene ID, top BLASTx hit, functional EggNOG annotation, and Gene Ontology (GO) term

Trinity Gene ID	BLASTx	EggNOG	GO term
TRINITY_DN470707_c0	Mini zinc finger protein 2	ZF-HD homeobox protein At4g24660-like	Cellular component: cytoplasm
TRINITY_DN477905_c1	-	-	-
TRINITY_DN483040_c2	Alkane hydroxylase MAH1	Cytochrome p450	Cellular component: endoplasmic reticulum
TRINITY_DN484423_c0	Chaperone protein dnaJ 8	ATP binding to DnaK	Cellular component: chloroplast
TRINITY_DN487264_c3	Geraniol 8-hydroxylase	-	Cellular component: endoplasmic reticulum membrane
TRINITY_DN490105_c1	Stigma-specific STIG1-like protein 4	Stigma-specific protein, Stig1	-
TRINITY_DN492051_c0	Pectinesterase	-	Cellular component: cell wall
TRINITY_DN493099_c2	-	-	-
TRINITY_DN495991_c3	Polygalacturonase	-	Cellular component: cell wall
TRINITY_DN496380_c4	Flowering-promoting factor 1	Positive regulation of flower development	-
TRINITY_DN498838_c0	Bergaptol O-methyltransferase	-	Molecular function: 5-hydroxyfuranocoumarin 5-O-methyltransferase activity
TRINITY_DN499610_c2	Tyrosine/DOPA decarboxylase 5	-	Molecular function: aromatic-L-amino acid decarboxylase activity
TRINITY_DN501923_c3	Sucrose synthase	Glycosyl transferase	Molecular function: sucrose synthase
TRINITY_DN502563_c3	(R)-mandelonitrile lyase-like	Oxidoreductase	Molecular function: flavin adenine dinucleotide binding
TRINITY_DN502958_c1	-	-	-

**Fig. 5:**
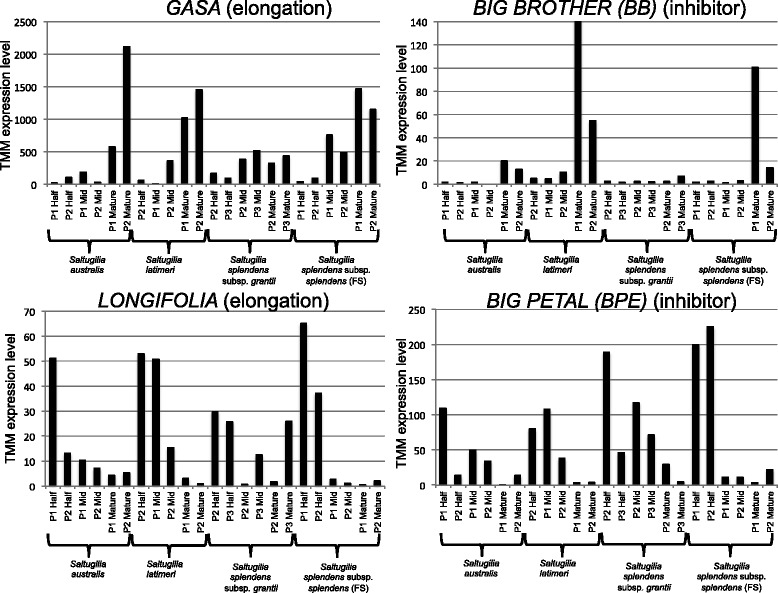
TMM expression levels of four previously annotated candidate genes, *GASA* (elongation)*, BIG BROTHER* (inhibitor)*, LONGIFOLIA* (elongation)*,* and *BIG PETAL* (inhibitor) showing differential expression between stages of development in *S. australis, S. latimeri, S. splendens* subsp. *grantii*, and *S. splendens* subsp. *splendens* (FS)

Homologs of many of the established candidate genes were annotated in the reference assembly, though only four of these showed differential expression between developmental stages. Homologs of *BIG BROTHER* and *GASA* showed upregulation in mature flowers compared to half-stage flowers, while homologs of *LONGIFOLIA* and *BIG PETAL* were upregulated in half-stage flowers compared to mature flowers (Fig. 5). Both *LONGIFOLIA* and *BIG PETAL* are more highly expressed in half-stage flowers than in any other stages, with *BIG PETAL* appearing to be expressed at higher levels in early flowers of the larger-flowered *S. splendens* subsp. *grantii* and *S. splendens* subsp. *splendens* (FS)*.* The other two candidate genes showing differential expression, *GASA* and *BIG BROTHER*, show higher expression in the mature flowers than any other stages. Comparisons between taxa do not show a clear trend, although *BIG BROTHER* appears to be expressed at higher levels in the field samples than in the greenhouse-grown plants.

The comparisons of the mature-stage flowers in the small- and large-flowered taxa resulted in 217 transcripts upregulated in the large-flowered taxa, with 100 transcripts having functional annotation. These include seven unigenes associated with cell wall modification and maintenance, including a polygalacturonase inhibitor and pectinesterase inhibitor. All of the cell wall unigenes appear to be upregulated in one of the two large-flowered species, mostly *S. splendens* subsp. *grantii*. However, two unigenes, a putative *beta-D-xylosidase* and *EXORDIUM*-like 2, are upregulated in both large-flowered taxa and may be associated with the observed larger flowers, with these transcripts functioning in secondary cell wall formation, as well as cell expansion. Additionally, all of the previously mentioned candidate genes from either *Arabidopsis* annotations or from previously hypothesized organ size pathways appear to show differences in mean expression between the large- and small-flowered taxa, but the differences were not significant, with an adjusted *p*-value of 0.997. The small-flowered taxa show 251 transcripts upregulated, with 121 of these transcripts annotated. The annotated transcripts include 12 unigenes associated with cell wall modification and maintenance and one repressor of early auxin response genes, but no unigenes associated with gibberellin activity.

## Discussion

### Phylogenetic relationships

*Saltugilia* has long been taxonomically difficult, with previous phylogenetic studies using a combination of sampling approaches, including a small number of loci and one individual per taxon [66], a small number of loci and many individuals per taxon [39], or many loci but only one individual per taxon [66]. The recently published HybPiper [42] used in this study is well suited for assembling contigs from hybrid-enriched data. Previous analyses used up to 90 loci [23], but these matrices included 37.2% missing data and many ambiguities (e.g. R, G, Y). Here only 54 loci were utilized, reducing the amount of missing data from 37.2 to 20.67%, even though missing data typically have been shown not to alter relationships substantially [67–69].

Previous studies of *Saltugilia* have reconstructed different relationships, with incongruences observed between nuclear and plastid data [23, 39, 66]. In our analyses based on 80 plastid protein-coding genes, *S. australis* is sister to *S. latimeri*. This relationship was also recovered by Weese and Johnson [39] in trees based on combined nuclear and plastid genes and may result from natural hybridization events between *S. australis* and *S. latimeri,* which can produce vigorous hybrids [38, 40]. Both nuclear and plastid data show a well-supported relationship between *S. splendens* subsp. *grantii* and *S. splendens* subsp. *splendens* (FS), with the addition of *S. caruifolia* in the clade. The placement of *S. splendens* subsp. *splendens* (GH) outside of *Saltugilia* is supported by multiple lines of evidence, including cell morphology comparisons [23], gross flower morphology, and transcriptome comparisons. From the inferred phylogeny of nuclear genes, based on both concatenated and species tree analyses, there appears to be a single transition from smaller flowers (*S. australis* and *S. latimeri*) to the larger flowers of *S. splendens* and *S. caruifolia*.

### Transcriptome comparisons and annotation

The filtered transcriptomes for each taxon contained between 51,020 and 92,672 Trinity genes. This is on par with recent transcriptome studies of flowers showing a range of 56,791 to 83,580 unigenes [14–16, 20, 21, 70]. The estimated number of genes for any transcriptome varies, with an average of 40,901 genes in a variety of tissues of *Glycine max* (soybean) and an average of 21,492 genes in *Zea mays* (corn) [71]*.* The estimated number of genes also varies by tissue type, with blueberry (*Vaccinium* section *Cyanococcus*) expressing 27,483 unigenes in leaves, 66,610 unigenes in berries, and 72,754 unigenes in developing flower buds [70].

Fewer than 25% of all transcripts in the *Saltugilia* reference were annotated, while other studies have shown a third or more of the transcriptome profile being unannotated [72–74]. The poor annotation for *Saltugilia* is likely due to the lack of genomic resources for Polemoniaceae, with the closest sequenced genomes in members of Ericales, including *Actinidia chinensis* (kiwi), *Primula vulgaris* (primrose)*, Rhododendron williamsianum,* and *Vaccinium corymbosum* (blueberry) (CoGepedia; https://genomevolution.org), which have limited annotations. Of the total annotated transcripts, 16.8% are molecular function genes, 12.3% are cellular component genes, and 2.5% are biological process genes. Even though the molecular function genes form the largest category, the majority of transcripts that appear to be differentially expressed by at least a four-fold change are the cellular component genes (Additional file 4: Table S4).

### Differential expression

Most comparisons among the three developmental stages in all six taxa resulted in differentially expressed transcripts with at least a four-fold difference. The greatest number of differentially expressed transcripts occurred between stages in the two taxa collected in the field, *S. latimeri* and *S. splendens* subsp. *splendens* (FS), which is consistent with studies in natural settings finding novel expression in otherwise silent genes relative to studies in controlled environments [13]. In general, more transcripts were differentially expressed between the mature and the half-stage flowers than between either of the other two developmental stages. The large number of differentially expressed genes is associated with increased cell size between the half-stage and mature flowers [23]. During development of any given taxon, the number of differentially expressed transcripts between the half-stage and mature flowers ranged from four to 418, excluding the 7182 transcripts from *S. splendens* subsp. *splendens* (FS). When all taxa were used as biological replicates for each of three stages, 784 transcripts were differentially expressed between the half and mature stages, with 514 transcripts upregulated in half-stage flowers and 270 transcripts upregulated in mature flowers. The observed increase in differentially expressed transcripts supports the claim that discovery of differentially expressed genes is easier with increased biological replicates, even at the cost of lower depth of sequencing [49].

The four unigenes highlighted in Fig. 4 appear to have direct implications for differences in flower size in *Saltugilia* based on their functional annotations and hypothesized organ size pathways [35]. In this pathway, organ growth occurs via cell elongation and/or cell proliferation, with cell proliferation directly influenced by growth-promoting factors. In *Saltugilia*, we found a homolog of *FLOWERING-PROMOTING FACTOR 1*, which has been shown to be involved in gibberellin signaling and may lead to enhanced responsiveness to gibberellins [75], with additional implications for changes in epidermal cell shape and formation of trichomes of leaves in overexpression studies [76]. The cell wall gene *PECTINESTERASE* has previously been shown to play a role in cell wall extension [77, 78]. The second cell wall gene, *POLYGALACTURONASE*, also known as *pectin depolymerase*, has been shown to weaken the pectin network, which gives strength to plant cell walls [79, 80]. Observed expression of *POLYGALACTURONASE* in mature flowers is associated with the formation of jigsaw cells [23], which require cell wall remodeling involving both pectin and cellulose [81]. The increased expression of *SUCROSE SYNTHASE* is important given that sucrose has been found to be abundant in flower petal tissue and is the first regulatory enzyme leading to starch biosynthesis in sucrose sink tissues (i.e. petals) [82, 83].

Many candidate genes associated with flower size have been identified in *Arabidopsis*. Putative networks and relationships among many of these have been reviewed previously [35, 36], yet most of these genes have not been investigated beyond model systems. Four of these genes, *BIG BROTHER*, *GASA, LONGIFOLIA*, and *BIG PETAL*, show differential expression between developmental stages in *Saltugilia*. *BIG BROTHER* has previously been shown to be a repressor of organ size by restricting the duration of cell proliferation [32, 36]. Taking into account that early growth of flowers primarily consists of cell division and later growth is due to cell expansion [84, 85], observing upregulation of *BIG BROTHER* in mature flowers is expected, given that the cells are primarily elongating at this stage. Previous analyses of *Saltugilia* suggest that by the half stage (50% anthesis) in flower development, increases in flower size are predominantly due to increases in cell size and not changes in cell number [23].

The identified member of the gibberellin-regulated gene family *GASA* showed higher expression in mature flowers than in any other stage. This pattern of expression is different than observed in *Arabidopsis*, where *GASA* showed higher expression in developing flower buds [26]. Later stages of flower development in *Saltugilia* are marked with increased cell elongation, especially in the elongated and jigsaw cells of the corolla tube [23]. *LONGIFOLIA* shows higher expression in half-stage flowers than in any other stage. Overexpression analyses of this gene in *Arabidopsis* resulted in elongated floral organs due to increased polar cell elongation rather than increased cell number [33]. The final candidate gene, *BIG PETAL*, exhibits higher expression in half-stage flowers. This pattern is opposite from expected, given that this gene restricts the expansion of petal cells in *Arabidopsis* [34]. The observation of the two identified repressor genes, *BIG BROTHER* and *BIG PETAL*, showing temporal differences in expression patterns is consistent when the cellular development of the flower is taken into consideration. With *BIG BROTHER* restricting cell division, higher expression in later stages is consistent with a pattern of cell elongation later in development. Likewise, *BIG PETAL* restricts cell elongation early in flower development when cell division is the primary driver of flower size differences.

All genes identified above fit into previously described organ size pathways [35]. Previous analyses of cell size show that early in development, cells increase in area, but change more substantially in overall shape, becoming less circular and more elongated [23]. Therefore, we hypothesize that *BIG PETAL* is inhibiting elongation of cells in the corolla tube, especially by changing these cells from elongated cells to jigsaw cells later in development, while *LONGIFOLIA* is acting on these same cells to make them less circular with a larger area. Later in development, *BIG PETAL* and *LONGIFOLIA* are downregulated, allowing cells to expand with the increased expression of *SUCROSE SYNTHASE, FLOWERING-PROMOTING FACTOR 1, GASA, PECTINESTERASE*, and *POLYGALACTURONASE. SUCROSE SYNTHASE* provides the sucrose necessary for cellular growth [86] and enhances the effects of *GASA* [27], as well as the targeted materials for *FLOWERING-PROMOTING FACTOR 1*. Higher expression of both *PECTINESTERASE* and *POLYGALACTURONASE* later in flower development may allow for the modification of the cell walls in the corolla tube to transition from elongated to jigsaw cells by breaking down sections of the cell wall for modification and laying down more pectin to strengthen the walls. During cell elongation, *BIG BROTHER* is also upregulated to cease cell division.

Incorporating phylogenetic relationships with differences in flower size suggests that larger flowers are the derived state in *Saltugilia* (Fig. 2). Only two of the eight candidate genes discussed above, *FLOWERING-PROMOTING FACTOR 1* and *BIG PETAL*, exhibit expression patterns expected based on the evolution of large flowers from small flowers. Along the phylogeny, there appear to be two transitions of increased expression of *FLOWERING-PROMOTING FACTOR 1*, with an increase between mature flowers of *S. australis* and *S. latimeri*, followed by a second transition between mature flowers of *S. latimeri* and mature flowers in the clade giving rising to *S. splendens* subsp. *grantii* and *S. splendens* subsp. *splendens* (FS) (Fig. 4). *BIG PETAL* follows a similar evolutionary trajectory, with a single increase in relative expression from the small-flowered taxa to the large-flowered taxa. This same trajectory is also observed in *Beta-D-xylosidase* and *EXORDIUM*-like 2, which show differential expression between the small- and large-flowered taxa.

## Conclusions

Four of the identified genes were previously characterized in *Arabidopsis*, with implications for floral organ size, while four others play a role in previously hypothesized pathways of mature organ size. Future functional analyses of these genes in non-model systems will help characterize the genetic control of differences in flower size. New phylogenetic relationships of *Saltugilia* are also presented based on current next-generation sequencing methods and recently developed approaches to phylogeny reconstruction. Few of the genes that are upregulated in mature relative to immature flowers also have higher mean expression levels in large-flowered versus small-flowered taxa, suggesting that the genetic control of changes in flower size during development may also govern interspecific differences in flower size, although these differences in mean expression levels are not significant. Further studies mapping shifts in gene expression to specific regions of the corolla should improve our ability to identify the interspecific differences in gene expression associated with flower size.

## Additional files

Additional file 1: Table S1. Phylogenetic information for each taxon, including number of nuclear and plastid genes, total bp for each data set, and SRA accession number. Voucher information denoted below (*) is from representatives of the geographic region sampled. (DOCX 104 kb)

Additional file 2: Table S2. Reported size of contig for each gene in the phylogenetic analysis for each taxon, including nuclear and plastid loci. (XLSX 64 kb)

Additional file 3: Table S3. Number of raw reads, cleaned paired-end reads, cleaned singleton reads, and SRA accession number for three developmental stages for each individual for each taxon, as well as summary from mapping the cleaned reads to the de novo assembled master reference including percent mapped, total Trinity genes, total transcripts, N50 value, median contig length, and average contig length. (DOCX 152 kb)

Additional file 4: Table S4. Comparison of developmental stages both within and between taxa, including the total number of upregulated transcripts, as well as the number of transcripts that were categorized as biological process, cellular component, molecular function, or unannotated. (DOCX 93 kb)

Additional file 5: Figure S1. Heatmap showing differential expression of transcripts between half, mid, and mature stages of development in each of the six taxa. Purple transcripts are downregulated, while yellow are upregulated. Cutoff values for differentially expressed transcripts were four-fold changes with a *p*-value less than 0.05. (PDF 1120 kb)

Additional file 6: Table S5. Differentially expressed transcripts from within and between taxa comparisons, with associated annotations from the BLASTx analysis. (XLSX 3293 kb)
