# Supplementary material for: Comparative transcriptomic analysis of the evolution and development of flower size in Saltugilia (Polemoniaceae)
Source: BMC Genomics. 2017 Jun 23;18:475. doi: 10.1186/s12864-017-3868-2 (PMC5481933; doi:10.1186/s12864-017-3868-2)
Supplement: Supplementary file 1 — Phylogenetic information for each taxon, including number of nuclear and plastid genes, total bp for each data set, and SRA accession number. Voucher information denoted below (*) is from representatives of the geographic region sampled. (DOCX 104 kb) [file 12864_2017_3868_MOESM1_ESM.docx]

Table S1. Phylogenetic information for each taxa, including number of nuclear and chloroplast genes, total bp for each data set, and SRA accession number for each taxa. Voucher information denoted below (*) were from representatives of the geographic region sampled.

| Species | Seed accession/Voucher | SRA accession | Nuclear genes | Total bp | Chloroplast genes | Total bp |
| --- | --- | --- | --- | --- | --- | --- |
| *Gilia brecciarum* | RSA705321* | SRR2658257 | 54 | 55,740 | 80 | 64,567 |
| *Gilia brecciarum subsp. brecciarum* | OGPC W6 30785 | SRR3542938 | 53 | 51,903 | 80 | 64,567 |
| *Gilia nevinii* | RSABG18895 | SRR3542940 | 53 | 48,850 | 77 | 64,210 |
| *Gilia stellata* | JOTR34200* | SRR2658258 | 54 | 53,565 | 80 | 64,567 |
| *Saltugilia australis* | L. Johnson Brigham Young University | SRR3542941 | 54 | 47,990 | 80 | 63,904 |
| *Saltugilia caruifolia* | RSABG19148 | SRR3691217 | 54 | 51,247 | 80 | 64,522 |
| *Saltugilia latimeri* | UCR261592* | SRR2658263 | 53 | 54,469 | 80 | 63,900 |
| *Saltugilia splendens subsp. grantii* | RSABG21757 | SRR3542942 | 45 | 29,203 | 78 | 63,780 |
| *Saltugilia splendens subsp. splendens* (GH) | RSABG22676 | SRR3542936 | 53 | 49,132 | 77 | 64,045 |
| *Saltugilia splendens subsp. splendens* (FS) | JOTR32513* | SRR2658260 | 54 | 54,517 | 80 | 64,522 |
| *Gymnosteris nudicaulis* | CAS635406 | SRR3540848 | 52 | 49,831 | 79 | 64,041 |
| *Gymnosteris parvula* (A03) | JEPS87405 | SRR3540872 | 52 | 49,521 | 77 | 62,688 |
| *Gymnosteris parvula* (A04) | UC300258 | SRR3540885 | 50 | 46,670 | 74 | 62,310 |
