# Supplementary material for: Comparative transcriptomic analysis of the evolution and development of flower size in Saltugilia (Polemoniaceae)
Source: BMC Genomics. 2017 Jun 23;18:475. doi: 10.1186/s12864-017-3868-2 (PMC5481933; doi:10.1186/s12864-017-3868-2)
Supplement: Supplementary file 3 — Number of raw reads, cleaned paired-end reads, cleaned singleton reads, and SRA accession number for three developmental stages for each individual for each taxon, as well as summary from mapping the cleaned reads to the de novo assembled master reference including percent mapped, total Trinity genes, total transcripts, N50 value, median contig length, and average contig length. (DOCX 152 kb) [file 12864_2017_3868_MOESM3_ESM.docx]

Table S3. Number of raw reads, cleaned paired-end reads, cleaned singleton reads, and SRA accession number for three developmental stages for each individual for each taxa, as well as summary from mapping the cleaned reads to the *de novo* assembled master reference including percent mapped, total trinity genes, total transcripts, N50 value, median contig length, and average contig length.

| Sample | Raw reads | Cleaned paired-end | Cleaned singletons | Percent mapped | Trinity genes | Total Transcripts | N50 | Median Contig | Average Contig | SRA number |
| --- | --- | --- | --- | --- | --- | --- | --- | --- | --- | --- |
| *S. australis* P1 Half | 24,354,149 | 19,692,304 | 2,878,236 | 67.87 | 96,854 | 111,514 | 423 | 277 | 409.85 | SRR2658638 |
| *S. australis* P1 Mid | 11,350,146 | 8,897,771 | 1,493,736 | 68.64 | 69,124 | 78,101 | 399 | 279 | 395.74 | SRR2658639 |
| *S. australis* P1 Mature | 13,793,185 | 11,392,869 | 1,509,147 | 66.33 | 83,102 | 91,931 | 399 | 273 | 403.25 | SRR2658637 |
| *S. australis* P2 Half | 22,109,207 | 18,326,378 | 2,604,496 | 77.6 | 122,136 | 136,467 | 325 | 259 | 358.22 | SRR2658641 |
| *S. australis* P2 Mid | 21,787,712 | 17,446,419 | 2,988,121 | 85.74 | 69,572 | 80,818 | 451 | 292 | 427.38 | SRR2658643 |
| *S. australis* P2 Mature | 23,991,291 | 19,134,580 | 3,465,950 | 76.22 | 65,367 | 86,073 | 669 | 342 | 539.29 | SRR2658640 |
| *S. caruifolia* P2 Half | 13,063,194 | 11,550,741 | 1,064,992 | 66.44 | 48,034 | 62,465 | 524 | 323 | 489.55 | SRR3691216 |
| *S. caruifolia* P2 Mature | 14,694,826 | 13,022,201 | 1,182,438 | 74.37 | 40,721 | 54,577 | 441 | 305 | 422.08 | SRR3691279 |
| *S. caruifolia* P3 Half | 5,407,867 | 4,397,485 | 761,414 | 76.02 | 50,044 | 67,294 | 572 | 351 | 508.79 | SRR3691280 |
| *S. caruifolia* P3 Mid | 17,056,280 | 15,014,001 | 1,420,022 | 78.36 | 38,736 | 52,099 | 493 | 317 | 454.89 | SRR3691277 |
| *S. caruifolia* P4 Mature | 5,224,711 | 3,869,784 | 1,060,244 | 50.19 | 32,549 | 41,774 | 536 | 339 | 482.79 | SRR3691278 |
| *S. caruifolia* P4 Mid | 10,651,775 | 9,145,325 | 1,072,128 | 73.64 | 49,731 | 67,195 | 465 | 315 | 441.28 | SRR3691276 |
| *S. latimeri* P1 Mature | 23,333,829 | 20,891,338 | 1,714,653 | 80.83 | 54,227 | 76,433 | 671 | 351 | 547.6 | SRR3691265 |
| *S. latimeri* P1 Mid | 29,232,323 | 26,200,704 | 2,119,832 | 81.22 | 64,963 | 91,694 | 740 | 354 | 573.14 | SRR3691252 |
| *S. latimeri* P2 Half | 17,883,654 | 15,948,114 | 1,374,737 | 81.67 | 61,380 | 87,865 | 689 | 349 | 553.43 | SRR3691053 |
| *S. latimeri* P2 Mature | 27,833,420 | 24,883,394 | 2,076,577 | 80.14 | 54,714 | 78,462 | 677 | 347 | 548.45 | SRR3691254 |

Table 3. Continued.

| Sample | Raw reads | Cleaned paired-end | Cleaned singletons | Percent mapped | Trinity genes | Total Transcripts | N50 | Median Contig | Average Contig | SRA number |
| --- | --- | --- | --- | --- | --- | --- | --- | --- | --- | --- |
| *S. latimeri* P2 Mid | 20,518,159 | 18,344,539 | 1,527,278 | 80.77 | 56,795 | 80,513 | 626 | 343 | 525.72 | SRR3691243 |
| *S. splendens* subsp. *grantii* P2 Half | 22,030,353 | 18,009,618 | 2,400,127 | 68.75 | 61,207 | 77,693 | 555 | 321 | 485.24 | SRR2658698 |
| *S. splendens* subsp. *grantii* P2 Mature | 25,155,564 | 20,621,269 | 2,713,027 | 67.37 | 100,663 | 118,652 | 476 | 269 | 428.89 | SRR2658705 |
| *S. splendens* subsp. *grantii* P2 Mid | 20,709,406 | 16,853,918 | 2,376,180 | 69.26 | 78,626 | 96,924 | 562 | 303 | 481.11 | SRR2658676 |
| *S. splendens* subsp. *grantii* P3 Half | 21,490,395 | 17,242,375 | 3,016,948 | 79.53 | 64,423 | 79,474 | 503 | 298 | 453.03 | SRR2658729 |
| *S. splendens* subsp. *grantii* P3 Mature | 24,568,612 | 20,528,235 | 2,651,066 | 76.86 | 119,375 | 132,012 | 323 | 253 | 361.52 | SRR2658736 |
| *S. splendens* subsp. *grantii* P3 Mid | 21,777,608 | 17,434,623 | 2,858,406 | 78.66 | 60,437 | 78,325 | 565 | 322 | 491.71 | SRR2658678 |
| *S. splendens* subsp. *splendens* (GH) P2 Half | 12,917,956 | 11,370,552 | 1,087,744 | 61.89 | 64,943 | 84,120 | 501 | 330 | 457.51 | SRR3691215 |
| *S. splenden*s subsp. *splendens* (GH) P2 Mature | 8,196,481 | 7,120,974 | 757,514 | 63.98 | 51,321 | 64,841 | 494 | 341 | 455.52 | SRR3691241 |
| *S. splendens* subsp. *splendens* (GH) P2 Mid | 8,211,501 | 7,246,205 | 678,156 | 58.42 | 51,786 | 66,814 | 488 | 333 | 452 | SRR3691224 |
| *S. splendens* subsp. *splendens* (GH) P3 Half | 6,962,046 | 6,054,463 | 653,793 | 61.98 | 56,688 | 72,005 | 493 | 335 | 455.64 | SRR3691242 |

Table 3. Continued.

| Sample | Raw reads | Cleaned paired-end | Cleaned singletons | Percent mapped | Trinity genes | Total Transcripts | N50 | Median Contig | Average Contig | SRA number |
| --- | --- | --- | --- | --- | --- | --- | --- | --- | --- | --- |
| *S. splendens* subsp. *splendens* (GH) P3 Mature | 8,384,307 | 7,340,858 | 762,475 | 59.03 | 58,205 | 76,037 | 477 | 327 | 441.9 | SRR3691225 |
| *S. splendens* subsp. *splendens* (GH) P3 Mid | 5,090,422 | 4,341,652 | 554,161 | 60.58 | 38,537 | 48,596 | 585 | 384 | 521.02 | SRR3691223 |
| *S. splendens* subsp. *splendens* (FS) P1 Half | 24,252,532 | 21,713,948 | 1,799,918 | 79.98 | 65,626 | 95,255 | 687 | 344 | 550.82 | SRR3691054 |
| *S. splendens* subsp. *splendens* (FS) P1 Mature | 19,328,429 | 17,399,422 | 1,360,560 | 80.09 | 51,058 | 72,591 | 639 | 339 | 529.36 | SRR3696527 |
| *S. splendens* subsp. *splendens* (FS) P1 Mid | 29,800,405 | 26,230,230 | 2,529,310 | 81.46 | 56,715 | 82,320 | 704 | 346 | 556.84 | SRR3696547 |
| *S. splendens* subsp. *splendens* (FS) P2 Half | 29,132,968 | 26,112,459 | 2,141,311 | 80.19 | 65,035 | 94,136 | 687 | 344 | 551.3 | SRR3696526 |
| *S. splendens* subsp. *splendens* (FS) P2 Mature | 27,300,922 | 24,395,012 | 2,055,253 | 79.7 | 55,979 | 80,384 | 672 | 342 | 544.09 | SRR3696531 |
| *S. splendens* subsp. *splendens* (FS) P2 Mid | 26,667,387 | 24,013,381 | 1,865,056 | 80.7 | 57,476 | 83,095 | 676 | 344 | 547.25 | SRR3696548 |
