# Supplementary material for: Comparative transcriptomic analysis of the evolution and development of flower size in Saltugilia (Polemoniaceae)
Source: BMC Genomics. 2017 Jun 23;18:475. doi: 10.1186/s12864-017-3868-2 (PMC5481933; doi:10.1186/s12864-017-3868-2)
Supplement: Supplementary file 4 — Comparison of developmental stages both within and between taxa, including the total number of upregulated transcripts, as well as the number of transcripts that were categorized as biological process, cellular component, molecular function, or unannotated. (DOCX 93 kb) [file 12864_2017_3868_MOESM4_ESM.docx]

Table S4. Comparison of developmental stages both within and between taxa, including the total number of upregulated transcripts, as well as the number of transcripts that were categorized as biological process, cellular component, molecular function, or unannotated.

| Comparison | Up-regulated transcriptome | Biological process | Cellular component | Molecular function | Unannotated | Total DE |
| --- | --- | --- | --- | --- | --- | --- |
| Sa Mature vs Sa Half | Mature | 0 | 39 | 14 | 27 | 80 |
|  | Half | 1 | 160 | 11 | 159 | 331 |
| Sa Mature vs Sa Mid | Mature | 2 | 19 | 26 | 12 | 59 |
|  | Mid | 2 | 91 | 4 | 79 | 176 |
| Sa Mid vs Sa Half | Mid | 0 | 4 | 0 | 2 | 6 |
|  | Half | 0 | 0 | 0 | 0 | 0 |
| Sc Mature vs Sc Mid | Mature | 0 | 0 | 0 | 0 | 0 |
|  | Mid | 0 | 0 | 0 | 0 | 0 |
| Sc Mature vs Sc Half | Mature | 0 | 10 | 9 | 66 | 85 |
|  | Half | 0 | 39 | 3 | 37 | 79 |
| Sc Mid vs Sc Half | Mid | 0 | 4 | 0 | 9 | 13 |
|  | Half | 0 | 0 | 0 | 4 | 4 |
| Sg Mature vs Sg Mid | Mature | 0 | 0 | 0 | 0 | 0 |
|  | Mid | 0 | 0 | 0 | 0 | 0 |
| Sg Mature vs Sg Half | Mature | 0 | 1 | 0 | 0 | 1 |
|  | Half | 0 | 1 | 0 | 2 | 3 |
| Sg Mid vs Sg Half | Mid | 0 | 0 | 0 | 0 | 0 |
|  | Half | 0 | 0 | 0 | 0 | 0 |
| Sl Mature vs Sl Mid | Mature | 2 | 89 | 20 | 71 | 182 |
|  | Mid | 1 | 474 | 105 | 270 | 850 |
| SsGH Mature vs SsGH Mid | Mature | 0 | 21 | 0 | 26 | 47 |
|  | Mid | 1 | 180 | 27 | 169 | 377 |
| SsGH Mature vs SsGH Half | Mature | 0 | 41 | 40 | 279 | 360 |
|  | Half | 0 | 27 | 4 | 27 | 58 |
| SsGH Mid vs SsGH Half | Mid | 0 | 1 | 1 | 5 | 7 |
|  | Half | 0 | 90 | 10 | 24 | 124 |

Table 6-4. Continuted.

| Comparison | Up-regulated transcriptome | Biological process | Cellular component | Molecular function | Unannotated | Total DE |
| --- | --- | --- | --- | --- | --- | --- |
| SsFS Mature vs SsFS Mid | Mature | 1 | 80 | 52 | 78 | 211 |
|  | Mid | 1 | 75 | 12 | 27 | 115 |
| SsFS Mature vs SsFS Half | Mature | 31 | 1,596 | 497 | 1,439 | 3,563 |
|  | Half | 17 | 2,256 | 232 | 1,114 | 3,619 |
| SsFS Mid vs SsFS Half | Mid | 22 | 731 | 223 | 536 | 1,512 |
|  | Half | 4 | 769 | 67 | 510 | 1,350 |
| All Mature vs All Mid | Mature | 0 | 0 | 1 | 2 | 3 |
|  | Mid | 0 | 23 | 1 | 18 | 42 |
| All Mature vs All Half | Mature | 6 | 121 | 70 | 73 | 270 |
|  | Half | 3 | 302 | 17 | 192 | 514 |
| All Mid vs All Half | Mid | 0 | 0 | 0 | 0 | 0 |
|  | Half | 0 | 0 | 0 | 0 | 0 |
