# Supplementary figures and images for: Comparative transcriptomic analysis of the evolution and development of flower size in Saltugilia (Polemoniaceae)
Source: BMC Genomics. 2017 Jun 23;18:475. doi: 10.1186/s12864-017-3868-2 (PMC5481933; doi:10.1186/s12864-017-3868-2)

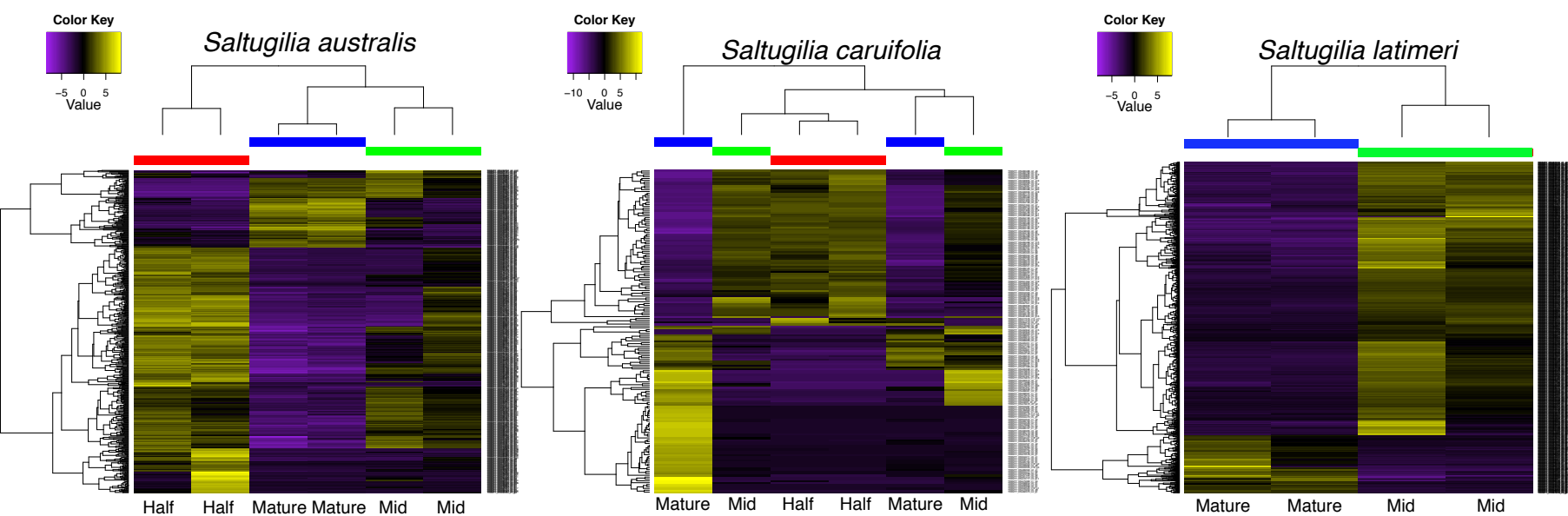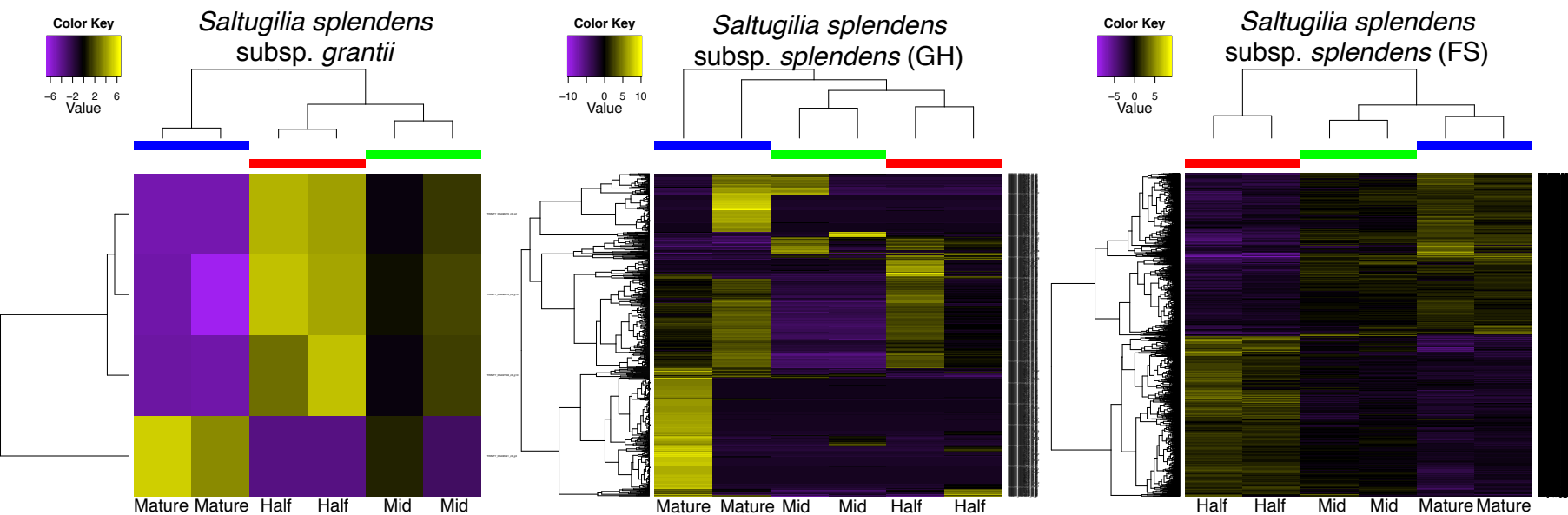

Mature  
Mid  
Half

Supplement: Supplementary file 5 — Heatmap showing differential expression of transcripts between half, mid, and mature stages of development in each of the six taxa. Purple transcripts are downregulated, while yellow are upregulated. Cutoff values for differentially expressed transcripts were four-fold changes with a p-value less than 0.05. (PDF 1120 kb) [file 12864_2017_3868_MOESM5_ESM.pdf]
